# The Impact of Sham Feeding with Chewing Gum on Postoperative Ileus Following Colorectal Surgery: a Meta-Analysis of Randomised Controlled Trials

**DOI:** 10.1007/s11605-019-04507-3

**Published:** 2020-02-26

**Authors:** Farah Roslan, Anisa Kushairi, Laura Cappuyns, Prita Daliya, Alfred Adiamah

**Affiliations:** 1 Gastrointestinal Surgery, Nottingham Digestive Diseases Centre and National Institute for Health Research (NIHR) Nottingham Biomedical Research Centre, Nottingham University Hospitals NHS Trust and University of Nottingham, Queen’s Medical Centre Campus, Derby Road, Nottingham, NG7 2UH UK; 2 East Midlands Surgical Academic Network (EMSAN), Queen’s Medical Centre Campus, Derby Road, Nottingham, NG7 2UH UK

**Keywords:** Sham feeding, Chewing gum, Postoperative ileus, Colorectal surgery

## Abstract

**Background:**

Chewing gum as a form of sham feeding is an inexpensive and well-tolerated means of promoting gastrointestinal motility following major abdominal surgery. Although recognised by the Enhanced Recovery After Surgery (ERAS) Society as one of the multimodal approaches to expedite recovery after surgery, strong evidence to support its use in routine postoperative practice is lacking.

**Methodology:**

A comprehensive literature review of all randomised controlled trials (RCTs) was performed in the Medline and Embase databases between 2000 and 2019. Studies were selected to compare the use of chewing gum versus standard care in the management of postoperative ileus (POI) in adults undergoing colorectal surgery. The primary outcome assessed was the incidence of POI. Secondary outcomes included time to passage of flatus, time to defecation, total length of hospital stay and mortality.

**Results:**

Sixteen RCTs were included in the systematic review, of which ten (970 patients) were included in the meta-analysis. The incidence of POI was significantly reduced in patients utilising chewing gum compared to those having standard care (RR 0.55, 95% CI 0.39, 0.79, *p* = 0.0009). These patients also had a significant reduction in time to passage of flatus (WMD − 0.31, 95% CI − 0.36, − 0.26, *p* < 0.00001) and time to defecation (WMD − 0.47, 95% CI − 0.60, − 0.34, *p* < 0.00001), without significant differences in the total length of hospital stay or mortality.

**Conclusion:**

The use of chewing gum after colorectal surgery is a safe and effective intervention in reducing the incidence of POI and merits routine use alongside other ERAS pathways in the postoperative setting.

## Introduction

Postoperative ileus (POI) is the temporary inhibition of gastrointestinal motility due to non-mechanical causes. Occurring after abdominal surgery, particularly after handling the bowel, it may result in nausea, vomiting and anorexia.^1^ Despite the lack of standard clinical definitions, the incidence of POI is reported to occur in up to 1 in 4 patients who have undergone gastrointestinal surgery making POI arguably the most frequent complication following digestive surgery.^2, 3^ Its sequelae include malnutrition, dehydration, electrolyte imbalance and aspiration pneumonia. POI poses a significant socioeconomic impact by prolonging hospital stay by as much as 5 days per patient and costing a staggering sum of 1.46 billion USD per annum to the health economy.^3, 4^ Surprisingly, not much progress has been made over the years in reducing its incidence and consequences.

The underlying mechanism of POI remains elusive; postulated as ensuing from surgical manipulation, intestinal oedema, electrolyte imbalance and medication such as opioids.^5^ The avoidance and management of risk factors can best be realised by application of multimodal pathways incorporating minimally invasive surgery, a stringent fluid regimen, the use of modern opioid-sparing pain strategies and early mobilisation.^5, 6^

Chewing gum, alongside other multipronged approaches recognised by the Enhanced Recovery After Surgery (ERAS) Society,^7^ is thought to simulate sham feeding which may stimulate gastrointestinal recovery postoperatively by the activation of the cephalic-vagal axis.^6, 8, 9^ This approach in sham feeding, is thought to encourage or initiate the processes involved in gut recovery without actually challenging the gut with food. Although early enteral feeding is recommended and widely practiced in the era of enhanced recovery, an estimated 20% of patients are unable to tolerate oral intake after the first postoperative day.^10, 11^ Additionally, many practitioners are reluctant to institute early feeding in some cases due to fears over safety and potential complications.^12^ Sham feeding could therefore be considered a safer alternative to early enteral nutrition in reducing POI.

Despite ERAS Society guidelines recommending the use of postoperative chewing gum to reduce POI, existing literature including a 2015 Cochrane review^13^ have been inconclusive in providing sufficient evidence for its use. Poor quality trials with small patient numbers, variation in the definition of POI, diverse perioperative care settings and heterogeneity in the operative procedures studied may all be contributing to the inconsistent evidence seen.

The aim of this meta-analysis is, therefore, to provide a valid and up-to-date summary of relevant high-quality trials comparing the impact of chewing-gum compared to standard care (the use of controls or placebos) in the management of POI in adults undergoing resectional large bowel surgery with or without an anastomosis. The primary outcome assessed was the incidence of POI. Secondary outcomes included time to passage of flatus, time to defecation, total length of hospital stay and mortality.

## Methodology

### Search Strategy

A comprehensive literature review of the Medline and Embase databases was conducted between 2000 and 2019. Search criteria were used to identify all studies evaluating the effect of postoperative chewing gum versus either a control or placebo on POI in patients undergoing open or laparoscopic colorectal surgery. The electronic search terms used were [“chewing gum” OR “sham feeding”] AND [“colorectal surgery”] AND [“postoperative ileus” OR “paralytic ileus”]. The Scottish Intercollegiate Guidelines Network (SIGN)^14^ filter was used to restrict studies to randomised controlled trials (RCTs). Only studies published in English and those involving adult patients over 16 years of age were included. Hand searches of the bibliographies of all included studies were performed to ensure comprehensive study inclusion. The meta-analysis was conducted according to the Preferred Reporting Items for Systematic Reviews and Meta-Analyses (PRISMA) statement.^15^

### Selection of Article

**Fig. 1:**
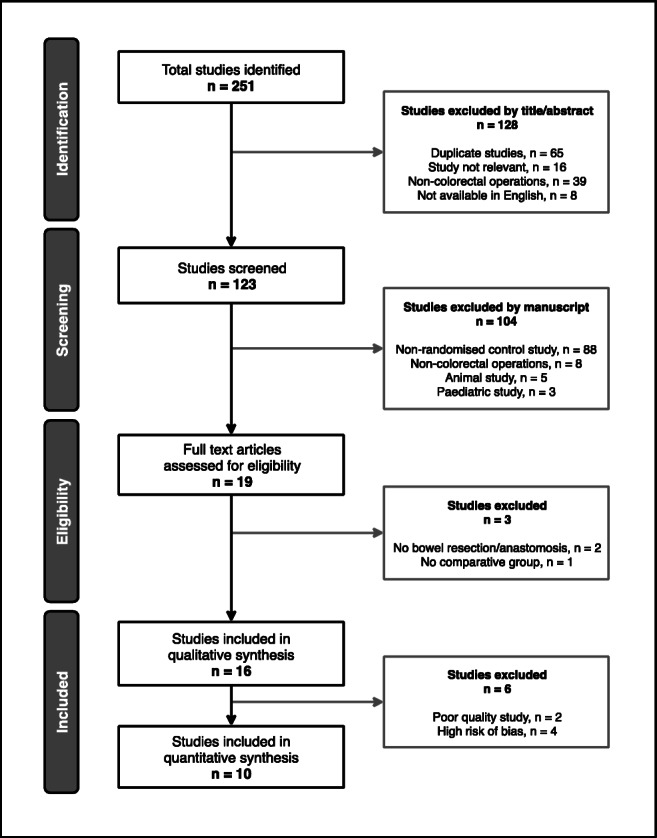
Preferred Reporting Items for Systematic reviews and Meta-Analysis (PRISMA) flow diagram

Preliminary exclusion was performed manually following title and abstract review (FR and AK) and subsequently through full-text review (AK, FR and AA) as illustrated in Fig. 1. “Colorectal surgery” included open, laparoscopic, emergency or elective procedures where the bowel was handled and resected with or without an anastomosis. Studies which involved patients primarily undergoing day-case or non-colorectal operations (upper gastrointestinal, hepatobiliary, bariatric, gynaecological, urological or vascular) were excluded, as were studies which did not report on any relevant clinical outcome measures, and studies where both patient groups received chewing gum. All except one study^16^ which reported on outcomes following mixed gastrointestinal surgery were excluded; this study was retained as colorectal and non-colorectal data were reported separately.^16^

### Data Extraction

Two independent authors (FR and AK) extracted data from the included studies according to predefined criteria. All RCTs were assessed for methodology, study design, inclusion and exclusion criteria, and outcome measures. Studies which presented their data as ‘median and range’ values were converted to ‘means and standard deviation (SD)’ data using the methods described by Hozo et al. and Wan et al. enabling all relevant data to be included in quantitative synthesis.^17, 18^

The methodological quality of each RCT was recorded for methods of randomisation, blinding, protocol violation and allocation concealment. All studies were scored using the Jadad scale.^19^ Any disagreement was resolved by consensus discussions with the senior member of the review team (AA). Data collected included type of surgery, execution of intervention and control measures, measured outcomes and statistically significant differences pertaining to chewing gum.

**Fig. 2:**
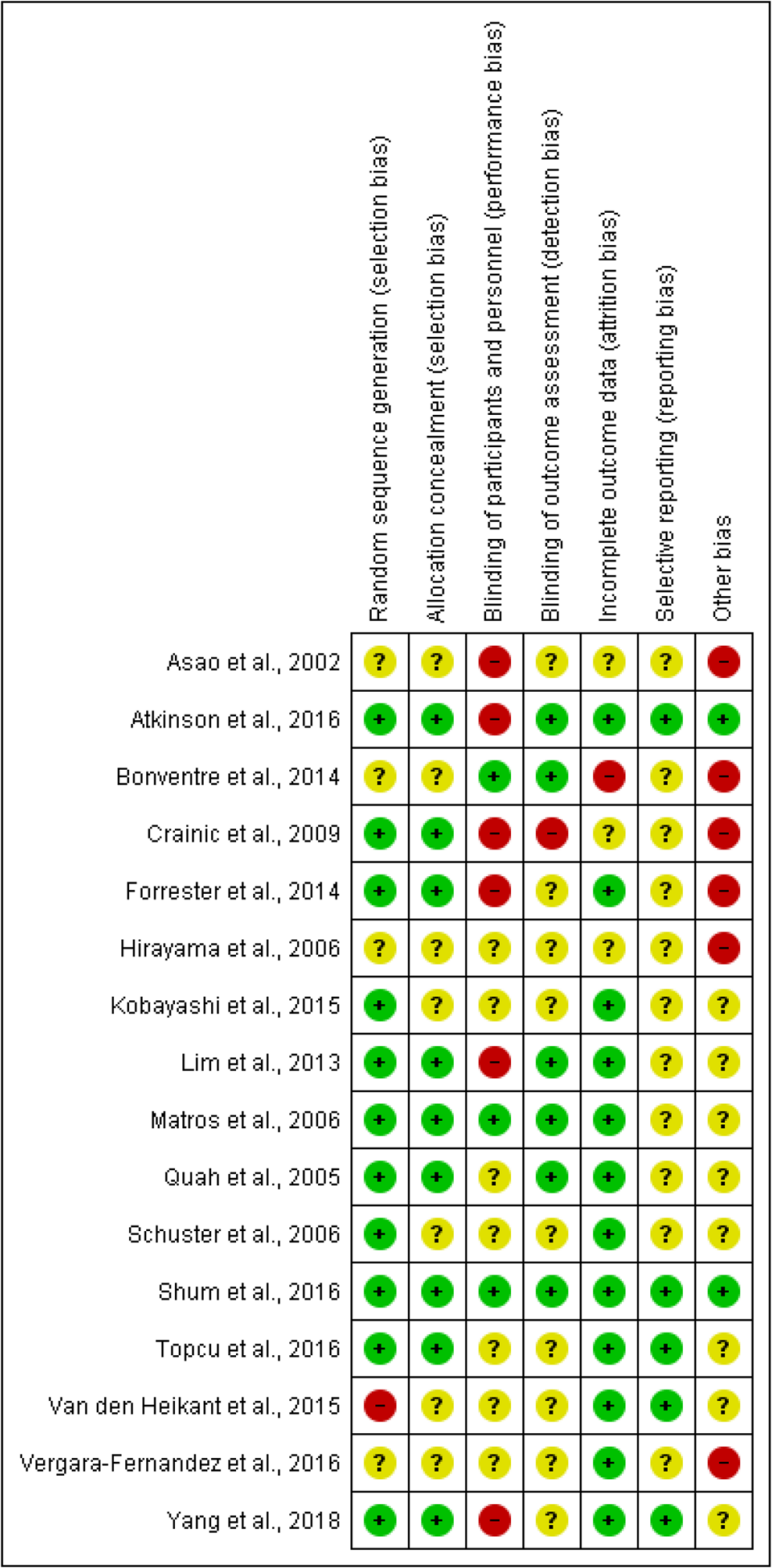
Summary of risk of bias assessment of the included studies

A risk of bias assessment was performed (PD, FR and AK) using the Cochrane Collaboration tool in RevMan 5.3,^20^ which focuses upon random sequence generation (selection bias), allocation concealment (selection bias), blinding of participants and personnel (performance bias), blinding of outcome assessment (detection bias), incomplete outcome data (attrition bias) and selective reporting (reporting bias). Each study was ranked as low, moderate or high risk of bias based on these criteria (Fig. 2).

### Statistical Analysis

Effect sizes for dichotomous outcomes were reported as risk ratio (RR) with 95% confidence intervals (CIs) and as weighted mean difference (WMD) for continuous data. Only studies of low to moderate risk of bias were included in quantitative pooling. Given the variability in the operative interventions, the different approaches (open versus laparoscopic) and the timings of chewing gum administration, it was decided a priori that a random effects model would be most appropriate for this meta-analysis.

Small studies with no power calculation (participant numbers less than 20), or those with high risk of bias were excluded from quantitative analysis to avoid overstating or understating the treatment effects. Statistical heterogeneity was assessed by the *I*^2^ statistic; threshold values of *I*^2^ equal to 25%, 50% and 75% represent low, moderate and high heterogeneity, respectively. These analyses were performed using RevMan 5.3 software (The Nordic Cochrane Center, The Cochrane Collaboration, Copenhagen, Denmark).^20^

### Protocol Registration

The study protocol for this meta-analysis was registered (CRD42018115852) with the PROSPERO database (www.crd.york.ac.uk/prospero).

## Results

### Study Quality

All selected studies included patients who underwent colorectal surgery. Table 1 summarises the characteristics of the sixteen included qualitative studies, ten of which were included in the quantitative synthesis.^21, 23, 25–29, 31–33^ A total of 970 patients undergoing colorectal surgery were randomised to either postoperative chewing gum (*n* = 481) or routine postoperative care (*n* = 489).

**Table 1:** The characteristics of included studies

Study	Surgical intervention (diagnosis)	Study intervention	Study control	Measured outcomes	Statistically significant difference with chewing gum
Asao et al. (2002)^12^	Elective laparoscopic colectomy (CRC)	Chewing gum [3× daily] (*n* = 10)	Standard care (*n* = 9)	A, B, C, M	Earlier time to flatus and time to defecation
Atkinson et al. (2016)^21^	Elective colorectal resection (CRC, UC, DD)	Chewing gum [10 min, 4× daily] (*n* = 199)	Standard care (*n* = 203)	A, C, D, G, H, I, J, N	None
Bonventre et al. (2014)^16^	Colorectal resection, Hartmann’s procedure [and Open gastrectomy and laparoscopic cholecystectomy]	A) Chewing gum [30 min, 3× daily] (*n* = 72) B) Olive oil supplement [2× daily] (*n* = 72) C) Chewing gum and olive oil supplement (*n* = 72) D) Water [2× daily] (*n* = 72)	Standard care (n = 72)	A, B, C	None for colorectal surgery Earlier time to defecation in gastrectomy patients
Crainic et al. (2009)^22^	Laparoscopic or exploratory colectomy	A) Chewing gum [30 min, 3× daily] (*n* = 20) B) Suck hard candy [3× daily] (*n* = 22)	Standard care (*n* = 24)	A, C, D	None
Forrester et al. (2014)^23^	Open or laparoscopic sigmoid/descending colon resection (CRC, DD)	Standard care and chewing gum [60 min, 3× daily] (*n* = 13)	A) Standard care (*n* = 17) B) Standard care and silicone patch [3× daily] (*n* = 17)	A, C, D, F, H	None
Hirayama et al. (2006)^24^	Elective laparotomy (CRC)	Standard care and chewing gum [30 min, 3× daily] (*n* = 10)	Standard care (*n* = 14)	A, B	Earlier time to flatus and time to defecation
Kobayashi et al. (2015)^25^	Left-sided colon resection (CRC)	Chewing gum [> 5 min, 3× daily] (*n* = 21)	Standard care (n = 22)	A, D, O	Longer colonic transit time Longer total LOS
Lim et al. (2013)^26^	Colorectal resection (any indication)	Chewing gum [15 min, 4× daily] (*n* = 77)	Standard care (*n* = 80)	A, C, D, H, K	None
Matros et al. (2006)^27^	Elective partial colectomy (CRC or benign disease)	A) Chewing gum [45 min, 3× daily] (*n* = 22) B) Acupressure bracelet [45 min, 3× daily] (*n* = 23)	Standard care (*n* = 21)	A, C, D, H	None
Quah et al. (2005)^28^	Open left-sided colonic resection (CRC)	Chewing gum [5 min, 3× daily] (*n* = 19)	Standard care (*n* = 19)	A, C, D, H	None
Shum et al. (2016)^29^	Laparoscopic colorectal resection (CRC)	Chewing gum [30 min, 3× daily] (*n* = 41)	Standard care (*n* = 41)	A, C, D, F, H	Earlier time to flatus, time to defecation and return of hunger
Schuster et al. (2006)^10^	Elective open sigmoid colectomy (CRC, DD)	Chewing gum [60 min, 3× daily] (*n* = 17)	Standard care (*n* = 17)	A, C, D, F, H	Earlier time to flatus and time to defecation Shorter total LOS
Topcu et al. (2016)^30^	Open colorectal surgery	Chewing gum [15 min, 3× daily] (*n* = 30)	Standard care (*n* = 30)	A, B, C, E	Earlier time to flatus, time to defecation, & resumption of diet Shorter total LOS
Van den Heijkant et al. (2015)^31^	Elective open colorectal resection	Chewing gum [duration and frequency not standardised] (*n* = 58)	Dermal patch [until enteral nutrition] (*n* = 62)	A, B, C, G	Lower rate of POI Shorter total LOS
Vergara-Fernandez et al. (2016)^32^	Elective colorectal surgery (CRC or benign disease)	Chewing gum [15 min, 4× daily] (*n* = 32)	Standard care (*n* = 32)	A, C, E, G, H	Earlier time to flatus Lower rate of POI and vomiting
Yang et al. (2018)^33^	Elective open proctectomy (CRC)	Chewing gum [30 min, 3× daily] (*n* = 43)	Standard care (*n* = 46)	A, B, G	Shorter time to first defecation Lower incidence of POI

A = time to flatus; B = time to defecation; C = length of hospital stay; D = time to bowel movement; E = time to feeding; F = feeling of hunger; G = POI; H = postoperative complications such as nausea, vomiting, abdominal distension, infection, bleeding; I = mortality; J = first day of bowel sound; K = consumption or tolerance of solid food; L = consumption or tolerance to clear fluid; M = tolerance to gum chewing; N = quality of life; O = gut hormones, P = postoperative use of medication such as epidural analgesia, opioids, laxatives; Q = inflammatory parameters; R = vital signs

*CRC* colorectal cancer, *UC* ulcerative colitis, *DD* diverticular disease, *LOS* length of stay, *POI* postoperative ileus

### Qualitative Analysis

Overall, the findings from the qualitative analyses were mixed and inconclusive. Qualitative analysis saw no significant difference in the incidence of POI between the chewing gum and control groups.^12, 26^ Four studies saw no difference in time to passage of flatus in patients who had chewing gum and those in the control group,^10, 16, 21, 22^ while three studies found a significant reduction.^12, 24, 30^ Qualitative analysis of time to passage of stool saw no significant differences in three studies,^16, 22, 23^ while four studies showed significant reductions when using chewing gum.^10, 12, 24, 30^ In terms of length of hospital stay, two studies showed no significant reduction,^12, 16^ while two studies showed a significant reduction in length of stay.^10, 30^

### Primary Outcome

#### Incidence of Postoperative Ileus

**Fig. 3:**
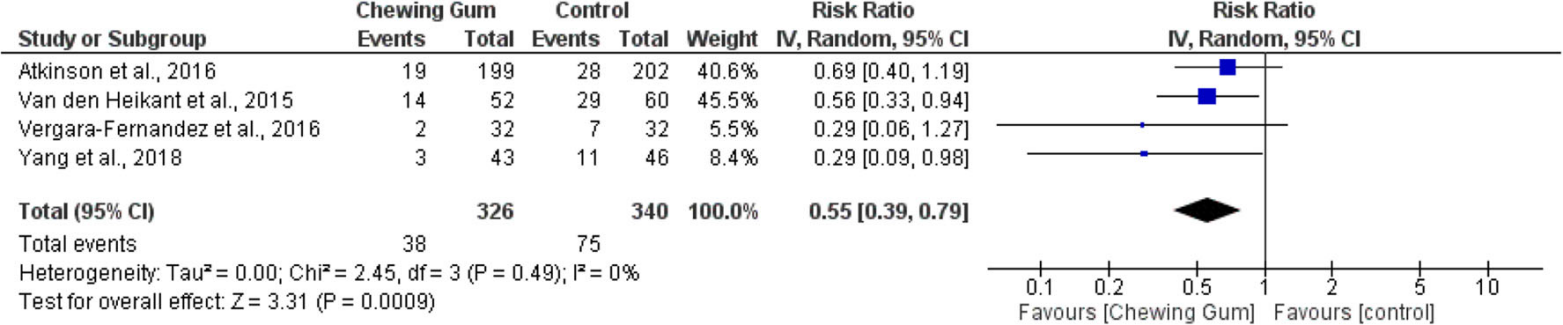
Incidence of postoperative ileus

In total, meta-analysis of four studies revealed 326 patients in the chewing gum group and 340 patients in the control group who showed significantly reduced incidence of POI (RR 0.55, 95% CI 0.39, 0.79, *p* = 0.0009, *I*^2^ = 0%) (Fig. 3).^21, 31–33^ Very low heterogeneity was observed in reporting the incidence of POI. The percentage of POI reported was lower in the gum-chewing arm ranging from 6 to 27%, whereas the control arm had a higher range between 14 and 48% (Table 3).

### Secondary Outcomes

#### Time to Passage of Flatus

**Fig. 4:**
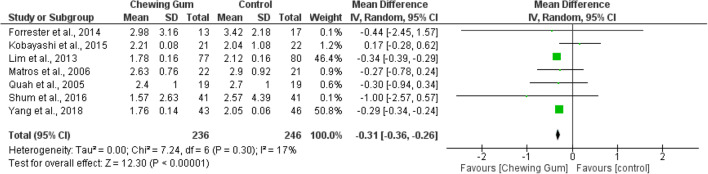
Time to passage of flatus

Seven RCTs (482 patients) were included in the quantitative meta-analysis of time to passage of flatus. Meta-analysis of 236 patients given chewing gum and 246 patients on placebo treatment showed significantly shorter time to passage of flatus in the chewing gum group (WMD − 0.31, 95% CI − 0.36, − 0.26, *p* < 0.00001) (Fig. 4).^23, 25–29, 33^ Heterogeneity was low with an *I*^2^ value of 17%.

#### Time to Defecation

**Fig. 5:**
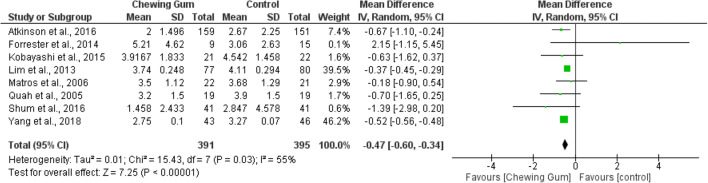
Time to defecation

Eight RCTs with 886 patients in total were included in the meta-analysis of time to defecation. Meta-analysis of 391 patients in the chewing gum group and 395 patients in the control group showed a significant decrease in time to defecation (data given in days) in the chewing gum group (WMD − 0.47, 95% CI − 0.60, − 0.34, *p* < 0.00001) (Fig. 5).^21, 23, 25–29, 33^ Heterogeneity was moderate at *I*^2^ of 55%.

#### Length of Hospital Stay

**Fig. 6:**
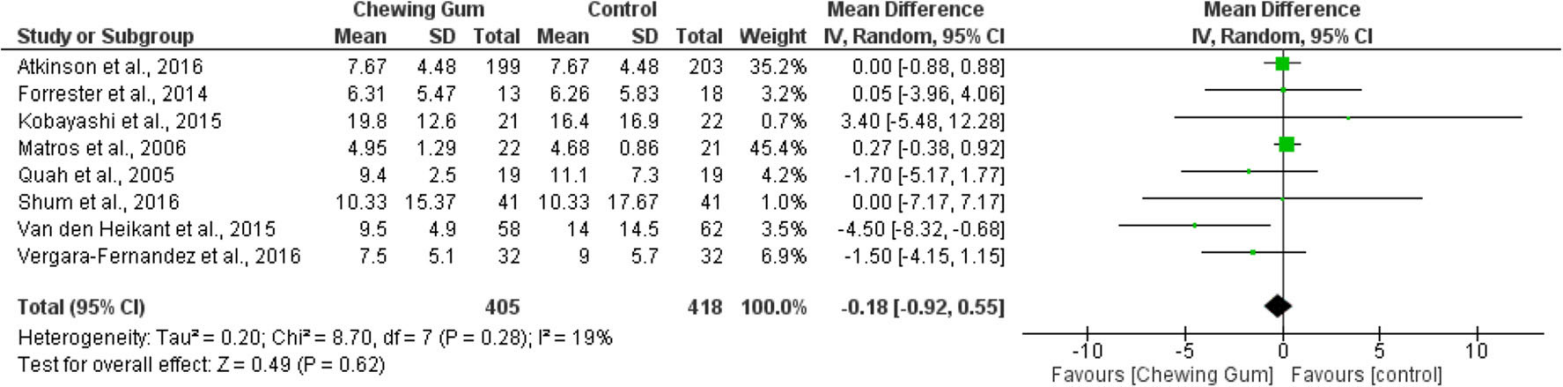
Length of hospital stay

Eight RCTs (823 patients) were included in the meta-analysis of length of hospital stay. Meta-analysis of 405 patients in the chewing gum group and 418 patients in the control group showed no significant reduction in length of stay (data presented in number of days) in the chewing gum group (WMD − 0.18, 95% CI − 0.92, 0.55, *p* = 0.28, *I*^2^ = 19%) (Fig. 6).^21, 23, 25, 27–29, 31, 32^

#### Mortality

**Fig. 7:**
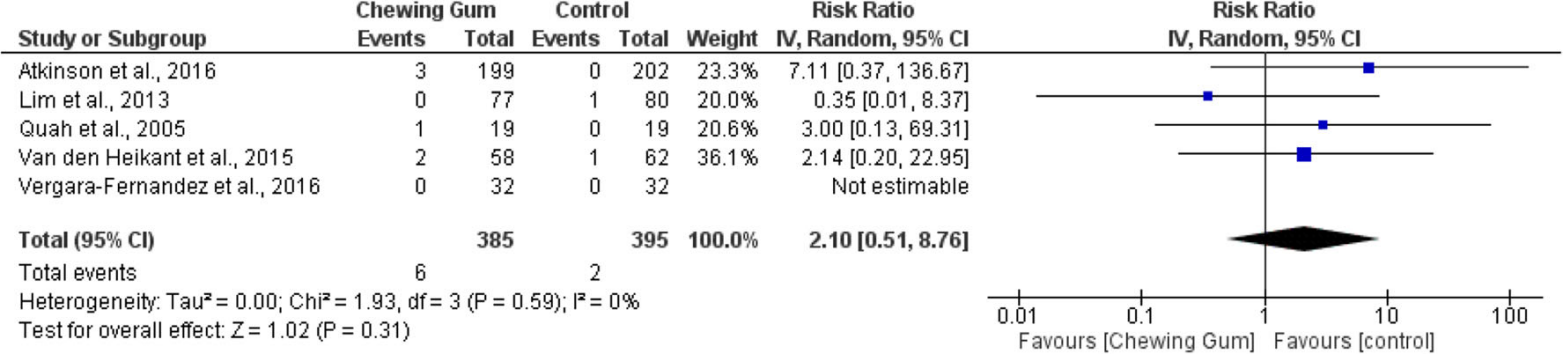
Mortality

Of the 16 included studies, five RCTs (780 patients) reported findings on patient mortality.^21, 26, 28, 31, 32^ Meta-analysis of 385 patients in the chewing gum group and 395 patients in the control group showed no significant difference in mortality between both groups (RR 2.10, 95% CI 0.51, 8.76, *p* = 0.59, *I*^2^ = 0%) (Fig. 7).

## Discussion

**Fig. 8:**
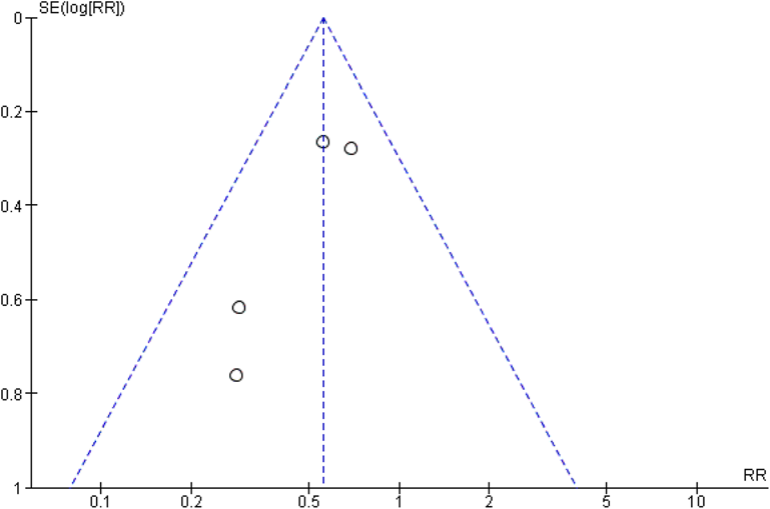
Funnel plot showing the assessment of publication bias

This systematic review of sixteen RCTs, of which ten were meta-analysed, comparing the efficacy of postoperative chewing gum against standard postoperative care demonstrated that chewing gum played a significant role in reducing the incidence of POI, time to passage of flatus and time to defecation in patients who had resectional large bowel surgery with or without an anastomosis. These findings were without any significant changes to total length of hospital stay or mortality (Fig. 8).

### Study Strengths

By selecting RCTs involving only adult patients who had undergone colorectal surgery meant that this systematic review and meta-analysis achieved a more homogenous group of patients in which to perform a quantitative analysis of outcomes. The removal of confounding factors and exclusion of patients with a smaller likelihood of ileus such as those undergoing day-case surgery also made the findings more relevant to the management of patients undergoing colorectal surgery.

In addition to outcomes explored in previous systematic reviews, we performed a meta-analysis on the incidence of POI and mortality. This paper is one of the few systematic reviews to directly explore the incidence of POI as the primary endpoint instead of the time to passage of flatus, which is the more commonly reported outcome in most RCTs. Our analysis demonstrated a significantly lower incidence of POI in patients who had chewing gum compared to patients who did not. In measuring the time to passage of flatus as the primary outcome, there is a potential for lack of accuracy in patient self-reporting and clinician documentation and this may explain the high heterogeneity and inconclusive findings reported in earlier meta-analyses.

Quality assessment revealed a wide variation between the included qualitative studies. A focus on moderate to high methodological quality studies, and those with a lower risk of bias was very important in formulating an accurate and relevant meta-analysis. In this review, we have managed to perform quantitative analysis of ten such relevant studies, which has resulted in a significant reduction of heterogeneity of the outcomes when compared to other meta-analyses on this topic.

### Limitations of Study

The major confounders that may have had an influence on the study outcomes include the type and duration of colorectal operations, minor variations of the definition of POI, outcome reporting and the temporal spread of studies in relation to the implementation of ERAS. However, after looking into descriptions of what constituted POI in each of the included studies (Table 2), we found that the four studies which reported on incidence of POI had relatively similar definitions which would limit the heterogeneity and minimise the potential for reporting bias.^21, 31–33^ This is also reflected in the significant and homogenous outcome of the primary endpoint.

**Table 2:** Definitions of postoperative ileus by study

Study	Definition of postoperative ileus used in study
Asao et al. (2002)^12^	None
Atkinson et al. (2016)^21^	Clinical state characterised by nausea, vomiting, abdominal distension and an inability to pass stools/flatus
Bonventre et al. (2014)^16^	A transient cessation of coordinated bowel motility after surgical intervention, which prevents effective transit of intestinal contents and/or tolerance of intake
Crainic et al. (2009)^22^	None
Forrester et al. (2014)^23^	Cessation of bowel function following surgery that lasts 3 days or longer
Hirayama et al. (2006)^24^	None
Kobayashi et al. (2015)^25^	None
Lim et al. (2013)^26^	None
Matros et al. (2006)^27^	Transient cessation of luminal transit after laparotomy or laparoscopy because of inhibition of intestinal motility
Quah et al. (2005)^28^	The delayed return of coordinated intestinal motility following abdominal surgery
Shum et al. (2016)^29^	None
Schuster et al. (2006)^10^	None
Topcu et al. (2016)^30^	None
Van den Heijkant et al. (2015)^31^	A lack of passage of flatus or stool and intolerance to oral intake for at least 24 h on day 5 postoperatively
Vergara-Fernandez et al. (2016)^32^	Absence of adequate bowel function on postoperative day 5, or the need for the insertion of a nasogastric tube because of abdominal distension, nausea and emesis after having started a liquid diet, in the absence of mechanical obstruction
Yang et al. (2018)^33^	Two or more episodes of nausea/vomiting, inability to tolerate oral diet over 24 h, absence of flatus over 24 h, distension and radiologic confirmation on or after postoperative day 4 without prior resolution

Nevertheless, there were also significant sources of heterogeneity amongst the studies including clinical factors such as the increasing implementation of ERAS protocols, and the impact of other postoperative complications unrelated to POI. Other sources of heterogenicity which have been well documented but difficult to quantify from the studies selected were non-clinical factors such as social care and occupational therapy.^34, 35^ These differences may be due to the geographical spread of the studies, or variation in operative management (Table 3).

**Table 3:** Summary of incidence of postoperative ileus, time to flatus and time to defecation across all studies

Study	Postoperative ileus incidence (*n*)				Time to flatus (days)				Time to defecation (days)			
	Intervention		Control		Intervention		Control		Intervention		Control	
	*n* (%)	Total	*n* (%)	Total	Mean ± SD	Total	Mean ± SD	Total	Mean ± SD	Total	Mean ± SD	Total
Asao et al. (2002)^12^	–	–	–	–	–	–	–	–	–	–	–	–
Atkinson et al. (2016)^21^	19 (10)	199	28 (14)	202	–	–	–	–	2 ± 1.50	159	2.67 ± 2.25	151
Bonventre et al. (2014)^16^	–	–	–	–	–	–	–	–	–	–	–	–
Crainic et al. (2009)^22^	–	–	–	–	–	–	–	–	–	–	–	–
Forrester et al. (2014)^23^	–	–	–	–	2.98 ± 3.16	13	3.42 ± 2.18	17	5.21 ± 4.62	9	3.06 ± 2.63	15
Hirayama et al. (2006)^24^	–	–	–	–	–	–	–	–	–	–	–	–
Kobayashi et al. (2015)^25^	–	–	–	–	2.21 ± 0.08	21	2.04 ± 1.08	22	3.92 ± 1.83	21	4.54 ± 1.46	22
Lim et al. (2013)^26^	–	–	–	–	1.78 ± 0.16	77	2.12 ± 0.16	80	3.74 ± 0.25	77	4.11 ± 0.29	80
Matros et al. (2006)^27^	–	–	–	–	2.63 ± 0.76	22	2.9 ± 0.92	21	3.5 ± 1.12	22	3.68 ± 1.29	21
Quah et al. (2005)^28^	–	–	–	–	2.4 ± 1	19	2.7 ± 1	19	3.2 ± 1.5	19	3.9 ± 1.5	19
Shum et al. (2016)^29^	–	–	–	–	1.57 ± 2.63	41	2.57 ± 4.39	41	1.46 ± 2.43	41	2.85 ± 4.58	41
Schuster et al. (2006)^10^	–	–	–	–	–	–	–	–	–	–	–	–
Topcu et al. (2016)^30^	–	–	–	–	–	–	–	–	–	–	–	–
Van den Heijkant et al. (2015)^31^	14 (27)	52	29 (48)	60	–	–	–	–	–	–	–	–
Vergara-Fernandez et al. (2016)^32^	2 (6)	32	7 (22)	32	–	–	–	–	–	–	–	–
Yang et al. (2018)^33^	3 (7)	43	11 (24)	46	1.76 ± 0.14	43	2.05 ± 0.06	46	2.75 ± 0.1	43	3.27 ± 0.07	46

– data not available

There was a large variation in the implementation of the intervention, in particular the duration and frequency of chewing gum administration in each of the studies. This ranged from as short as 5 min of chewing 3 times daily, to as long as 60 min of chewing 3 times daily. One of the studies did not standardise the duration or frequency of gum chewing by allowing patients to chew gum to their own liking.^31^

Another limitation of note is the temporal spread of the studies as perioperative practice has undergone many changes over the past 16 years and may well have created differences between the earliest study^12^ to the most recent study.^33^ Therefore ‘standard care’ received by patients in the control arm was probably not standardised between studies. The same undoubtedly applies for postoperative management algorithms where there has been a shift towards early resumption of oral intake, optimisation of fluid management, the selective use of anaesthesia, and the use of medications to reduce POI such as Alvimopan. Furthermore, none of the studies utilising ERAS pathways included an assessment of compliance with their ERAS standards. This is of significant relevance due to the positive correlation between compliance with standards and clinical outcomes. Additionally, given that some studies had not incorporated ERAS principles, the impact of chewing gum in a firm setting of enhanced recovery remains undefined.

Finally, one might argue against the effectiveness of chewing gum in reducing the occurrence of POI, as no significant reduction in the length of hospital stay was demonstrated. However, as our findings revealed that neither the time to passage of flatus nor the time to defecation was reduced by more than a day with the use of sham feeding with chewing gum, we did not expect a significant reduction in length of stay. Moreover, the various non-clinical factors which affect hospital discharge across different centres, in addition to non-standardised ERAS protocols would suggest that length of hospital stay may not be an accurate outcome indicator for measuring the effectiveness of chewing gum at reducing POI.^36–38^ It is therefore difficult to state that because length of hospital stay was unimproved, the impact of chewing gum is without merit, as our meta-analysis does demonstrate a significant reduction in the reporting of ileus, time to passage of flatus and time to passage of stool.

### Comparison with Other Studies

Our findings follow a similar trend to previous systematic reviews by Fitzgerald and Sua who found significant reduction in time to passage of flatus and time to bowel movement, and no significant difference in length of hospital stay and complication rates.^9, 39^ Since we would not expect any harm from chewing gum, it was not surprising to find no significant association with complication rates, reaffirming the safety of chewing gum use postoperatively.

Nonetheless, our findings diverge from other reviews with regard to length of stay. A 2015 Cochrane review^13^ demonstrated a significant reduction in time to first flatus, time to bowel movement and length of hospital stay in patients who had chewing gum compared to control groups, echoed by two other reviews.^8, 40^ However, it should be noted that this review included studies with paediatric patients, and patients undergoing gynaecological procedures, which might have affected the results.

### Health Policy Implications

Taking into account that POI is the most frequent complication after gastrointestinal surgery, any incremental benefit in reducing the duration of symptoms is likely to be beneficial overall. Chewing gum as an alternative to early enteral feeding is proven to be a safe and effective intervention. Administration of chewing gum on the first postoperative day could prevent POI and the morbidity associated with delayed gut motility.

## Conclusion

This meta-analysis of ten moderate to high quality RCTs provides evidence of the benefit of using sham feeding with chewing gum to reduce the incidence of POI in patients undergoing colorectal surgery. While the length of hospital stay is unimproved, there is clear reduction in the time to passage of flatus and time to defecation. Recognising that POI has a multifactorial underlying pathophysiology, chewing gum is unlikely to be the sole answer to the complex problem of POI in patients undergoing colorectal surgery. However, given the low side-effect profile, wide availability and patient acceptance of postoperative chewing gum use in addition to its potential benefit of reducing POI suggests it should be routinely considered as part of existing ERAS protocols.

## References

[1] Vather R, Trivedi S, Bissett I (2013). Defining Postoperative Ileus: Results of a Systematic Review and Global Survey. J Gastrointest Surg..

[2] Chapuis PH, Bokey L, Keshava A (2013). Risk factors for prolonged ileus after resection of colorectal cancer: An observational study of 2400 consecutive patients. Ann Surg..

[3] Asgeirsson T, El-Badawi K, Mahmood A, Barletta J, Luchtefeld M, Senagore AJ (2010). Postoperative Ileus: It Costs More Than You Expect. J Am Coll Surg..

[4] Goldstein JL, Matuszewski KA, Delaney CP, et al. Inpatient Economic Burden of Postoperative Ileus Associated with Abdominal Surgery in the United States. 2007;32(2).

[5] Venara A, Neunlist M, Slim K (2016). Postoperative ileus: Pathophysiology, incidence, and prevention. J Visc Surg..

[6] Gero D, Gié O, Hübner M, Demartines N, Hahnloser D (2017). Postoperative ileus: in search of an international consensus on definition, diagnosis, and treatment. Langenbeck’s Arch Surg..

[7] ERAS. http://erassociety.org/.

[8] Noble EJ, Harris R, Hosie KB, Thomas S, Lewis SJ (2009). Gum Chewing Reduces Postoperative Ileus? A Systematic Review and Meta-Analysis. Int J Surg..

[9] Su’a BU, Pollock TT, Lemanu DP, MacCormick AD, Connolly AB, Hill AG (2015). Chewing gum and postoperative ileus in adults: A systematic literature review and meta-analysis. Int J Surg..

[10] Schuster R, Grewal N, Greaney GC, Waxman K (2006). Gum chewing reduces ileus after elective open sigmoid colectomy. Arch Surg..

[11] Kristoffer L, Kjave Jorn FT (2008). Allowing Normal Food at Will After Major Upper Gastrointestinal Surgery Does Not Increase Morbidity: A Randomized Multicenter Trial. Ann Surg.

[12] Asao T, Kuwano H, Jichi N, Morinaga N, Hirayama I, Ide M (2002). Gum chewing enhances early recovery from postoperative ileus after laparoscopic colectomy. J Am Coll Surg..

[13] Short, Vanessa; Herbert, Georgia; Perry R. Chewing gum for postoperative recovery of gastrointestinal function. *Cochrane Database Syst Rev*. 2015;(February):1–221. https://www.cochranelibrary.com/cdsr/doi/10.1002/14651858.CD006506.pub3/full 10.1002/14651858.CD006506.pub3 PMC9913126 25914904

[14] SIGN search-filters-randomised-controlled-trials.

[15] Moher D, Liberati A, Tetzlaff J, Altman DG, The PRISMA Group (2009). PRISMA 2009 Flow Diagram. PLoS Med.

[16] Bonventre S, Inviati A, Di Paola V (2014). Evaluating the efficacy of current treatments for reducing postoperative ileus: A randomized clinical trial in a single center. Minerva Chir..

[17] Hozo SP, Djulbegovic B, Hozo I (2005). Estimating the mean and variance from the median, range, and the size of a sample. BMC Med Res Methodol..

[18] Wan X, Wang W, Liu J, Tong T. Estimating the sample mean and standard deviation from the sample size, median, range and/or interquartile range. 2014:1–13. 10.1186/1471-2288-14-135 PMC4383202 25524443

[19] Jadad AR, Moore RA, Carroll D, et al. Assessing the Quality of Reports of Ramdomized Clinical Trials: Is Blinding Necessary? 1996;17(January 1996):1–12. 10.1016/0197-2456(95)00134-4 10.1016/0197-2456(95)00134-4 8721797

[20] The Cochrane Collaboration. RevMan 5.3. 2017. https://community.cochrane.org/help/tools-and-software/revman-5/revman-5-download.

[21] Atkinson C, Penfold CM, Ness AR (2016). Randomized clinical trial of postoperative chewing gum versus standard care after colorectal resection. Br J Surg..

[22] Crainic C, Erickson K, Gardner J (2009). Comparison of methods to facilitate postoperative bowel function. Medsurg Nurs..

[23] Forrester DA (Tony), Doyle-Munoz J, McTigue T, DʼAndrea S, Natale-Ryan A. The Efficacy of Gum Chewing in Reducing Postoperative Ileus. *J Wound, Ostomy Cont Nurs*. 2014;41(3):227–232. 10.1097/WON.0000000000000019 10.1097/WON.0000000000000019 24621587

[24] Hirayama I, Suzuki M, Ide M, Asao T, Kuwano H (2006). Gum-chewing stimulates bowel motility after surgery for colorectal cancer. Hepatogastroenterology..

[25] Kobayashi T, Masaki T, Kogawa K, Matsuoka H, Sugiyama M (2015). Efficacy of Gum Chewing on Bowel Movement After Open Colectomy for Left-Sided Colorectal Cancer: A Randomized Clinical Trial. Dis Colon Rectum..

[26] Lim P, Morris OJ, Nolan G, Moore S, Draganic B, Smith SR (2013). Sham feeding with chewing gum after elective colorectal resectional surgery: A randomized clinical trial. Ann Surg..

[27] Matros E, Rocha F, Zinner M (2006). Does Gum Chewing Ameliorate Postoperative Ileus? Results of a Prospective, Randomized, Placebo-Controlled Trial. J Am Coll Surg..

[28] Quah HM, Samad A, Neathey AJ, Hay DJ, Maw A (2006). Does gum chewing reduce postoperative ileus following open colectomy for left-sided colon and rectal cancer? - A prospective randomized controlled trial. Color Dis..

[29] Shum NF, Choi HK, Mak JCK, Foo DCC, Li WC, Law WL (2016). Randomized clinical trial of chewing gum after laparoscopic colorectal resection. Br J Surg..

[30] Topcu SY, Oztekin SD (2016). Effect of gum chewing on reducing postoperative ileus and recovery after colorectal surgery: A randomised controlled trial. Complement Ther Clin Pract..

[31] Van Den Heijkant TC, Costes LMM, Van Der Lee DGC (2015). Randomized clinical trial of the effect of gum chewing on postoperative ileus and inflammation in colorectal surgery. Br J Surg..

[32] Vergara-Fernandez O, Gonzalez-Vargas AP, Castellanos-Juarez JC, Salgado-Nesme N, Sanchez-Garcia RE (2016). Usefulness of Gum Chewing to Decrease Postoperative Ileus in Colorectal Surgery with Primary Anastomosis: A Randomized Controlled Trial. Rev Invest Clin..

[33] Yang P, Long WJ, Li W. Chewing Xylitol Gum could Accelerate Bowel motility Recovery after Elective Open Proctectomy for Rectal Cancer. 2018:53–58. 10.24875/RIC.18002428 10.24875/RIC.18002428 29513303

[34] Aravani A, Samy EF, Thomas JD, Quirke P. A retrospective observational study of length of stay in hospital after colorectal cancer surgery in England (1998–2010). 2019;95(47):1–13. 10.1097/MD.0000000000005064 10.1097/MD.0000000000005064 PMC5134848 27893655

[35] Shepperd S, Parkes J, Jjm M, et al. Discharge planning from hospital to home (Review). 2009;(1):10–12. 10.1002/14651858.CD000313.pub2.Copyright 10.1002/14651858.CD000313.pub2 14973952

[36] Brasel K, Lim H, Nirula RWJ (2007). Length of stay - An Appropriate Quality Measure?. Arch Surg..

[37] Lingsma HF, Bottle A, Middleton S, Kievit J, Steyerberg EW (2018). Marang-Van De Mheen PJ. Evaluation of hospital outcomes: The relation between length-of-stay, readmission, and mortality in a large international administrative database. BMC Health Serv Res..

[38] Chapman SJ, Thorpe G (2019). Systematic review of definitions and outcome measures for return of bowel function after gastrointestinal surgery. BJS Open..

[39] Fitzgerald JEF, Ahmed I (2009). Systematic review and meta-analysis of Chewing-gum therapy in the reduction of postoperative paralytic ileus following gastrointestinal surgery. World J Surg..

[40] Zhang, Hui; Deng, Yong-Hong; Shuai, Ting; Song G-M. Chewing gum for postoperative ileus after colorectal surgery: A systematic review of overlapping meta-analyses. 2017;4(2):92–104. 10.1016/j.ecns.2009.08.002.https

